# Manzamine A reduces androgen receptor transcription and synthesis by blocking E2F8‐DNA interactions and effectively inhibits prostate tumor growth in mice

**DOI:** 10.1002/1878-0261.13637

**Published:** 2024-04-11

**Authors:** Dev Karan, Seema Dubey, Sumedha Gunewardena, Kenneth A. Iczkowski, Manohar Singh, Pengyuan Liu, Angelo Poletti, Yeun‐Mun Choo, Hui‐Zi Chen, Mark T. Hamann

**Affiliations:** ^1^ Department of Pathology, and MCW Cancer Center Medical College of Wisconsin Milwaukee WI USA; ^2^ Department of Cell Biology and Physiology University of Kansas Medical Center KS USA; ^3^ Department of Physiology and Center of Systems Molecular Medicine Medical College of Wisconsin Milwaukee WI USA; ^4^ Department of Pharmacological and Biomolecular Sciences University of Milan Italy; ^5^ Department of Chemistry University of Malaya Kuala Lumpur Malaysia; ^6^ Department of Medicine Medical College of Wisconsin Milwaukee WI USA; ^7^ Department of Drug Discovery and Biomedical Sciences and Public Health, Colleges of Pharmacy and Medicine, Hollings Cancer Center Medical University of South Carolina Charleston SC USA

**Keywords:** androgen receptor, E2F8, manzamine A, prostate cancer

## Abstract

The androgen receptor (AR) is the main driver in the development of castration‐resistant prostate cancer, where the emergence of AR splice variants leads to treatment‐resistant disease. Through detailed molecular studies of the marine alkaloid manzamine A (MA), we identified transcription factor E2F8 as a previously unknown regulator of *AR* transcription that prevents AR synthesis in prostate cancer cells. MA significantly inhibited the growth of various prostate cancer cell lines and was highly effective in inhibiting xenograft tumor growth in mice without any pathophysiological perturbations in major organs. MA suppressed the full‐length AR (AR‐FL), its spliced variant AR‐V7, and the AR‐regulated prostate‐specific antigen (*PSA*; also known as *KLK3*) and human kallikrein 2 (*hK2*; also known as *KLK2*) genes. RNA sequencing (RNA‐seq) analysis and protein modeling studies revealed E2F8 interactions with DNA as a potential novel target of MA, suppressing *AR* transcription and its synthesis. This novel mechanism of blocking AR biogenesis via E2F8 may provide an opportunity to control therapy‐resistant prostate cancer over the currently used AR antagonists designed to target different parts of the *AR* gene.

## Graphical abstract

This study used a marine sponge product, Manzamine A (MA), and identified E2F8 as a novel regulator of androgen receptor (AR) transcription. MA abrogated AR biogenesis and inhibited prostate tumor growth in mice. These findings demonstrated that as a small molecule inhibitor of E2F8, MA may serve as a potential lead compound to overcome therapy resistance to prostate cancer caused by AR remodeling.

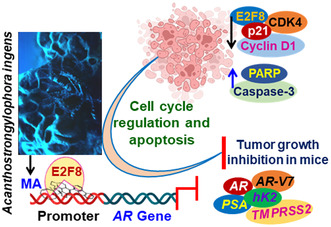

**Abbreviations**
ABC: ATP‐binding cassette
ActD: actinomycin D
ADT: androgen deprivation therapy
ALB: albumin
ALP: alkaline phosphate
ALT: alanine aminotransferase
ANOVA: analysis of variance
AR: androgen receptor
ARE: androgen response elements
AR‐FL: AR‐full length
AR‐Vs: AR splice variants
AST: aspartate aminotransferase
BMPR: bone morphogenetic protein receptor
CDK: cyclin‐dependent kinase
CHX: cycloheximide
CK: creatine kinase
CK2α: casein kinase alpha
CRPC: castration‐resistant prostate cancer
DBD: DNA binding domain
DEGs: differentially expressed genes
DMSO: dimethyl sulfoxide
GAPDH: glyceraldehyde 3‐phosphate dehydrogenase
GGT: gamma‐glutamyltransferase
GNA14: G‐protein alpha
GRPR: gastrin‐releasing peptide receptor
hK2: human kallikrein 2 (KLK2)
IC50: half‐maximal inhibitory concentration
KEGG: Kyoto Encyclopedia of Genes and Genomes
LBD: ligand‐binding domain
MA: manzamine A
MELK: maternal embryonic leucine zipper kinase
mRNA: messenger ribonucleic acid
MUC2: mucin 2
NaCl: sodium chloride
NTD: N‐terminal domain
PARP: poly (ADP‐ribose) polymerase
PBS: phosphate buffer saline
pCMV: plasmid cytomegalovirus
PDB: protein data bank
pGL: plasmid (luciferase reporter vector)
PHACTR3: phosphatase and actin regulator
PSA: prostate‐specific antigen
PTPN5: protein tyrosine phosphatase non‐receptor
qRT PCR: quantitative reverse transcriptase polymerase chain reaction
Rhiz: rhizochalinin
SINT1: sintokamide A
SIX1: sine oculis homeobox homolog 1
TP: total protein

## 1 Introduction

Prostate cancer is the most commonly diagnosed cancer and the second leading cause of cancer‐related deaths among men in the United States, accounting for an estimated 30 000 deaths every year [1]. Initially, prostate cancer cells depend on androgens for their survival, growth, and proliferation; hence androgen deprivation therapy (ADT) remains the first‐line approach to treatment and has become the standard of care [2, 3]. Unfortunately, following the initial therapy, ~ 20–30% of prostate cancer patients relapse with progressive disease and develop castration‐resistant prostate cancer (CRPC), which continues to evolve into metastatic disease. Several new drugs, including enzalutamide and darolutamide, have been approved for metastatic CRPC in past years, demonstrating positive responses and therapeutic improvements [4]. Nonetheless, the battle against prostate cancer is still hampered primarily due to the development of drug resistance and the risk of unwanted side effects ultimately leading to treatment failure.

The androgen receptor (*AR*) is vital to the development and progression of metastatic CRPC through *AR* reprogramming and maintains the growth of prostate cancer cells in an androgen‐depleted state [5, 6]. Prostate cancer cells express endogenous full‐length *AR* (AR‐FL), where the emergence of *AR* splice variants (AR‐Vs) is associated with the therapy‐resistant disease. The structural organization of *AR* has been studied extensively, and the N‐terminal domain (NTD) is known to function as a potent *AR* transcriptional activator. The evolved AR‐Vs lack ligand‐binding domain (LBD) and are constitutively active, driving androgen‐independent *AR* transcription, and is critical in developing treatment‐resistant prostate cancer [7, 8, 9]. Therefore, developing new therapeutic tools and identifying novel targets inhibiting *AR* activation and its regulated network remains an active area of prostate cancer research to block the development and progression of CRPC. An alternative approach to androgen depletion through castration or treatment with AR antagonists is to abrogate the AR synthesis through its upstream regulator E2F8, identified in this study. E2F8 is an atypical transcriptional factor of the E2F family and is known to repress E2F‐target gene expression independent of *RB* binding [10, 11]. Emerging evidence emphasized the oncogenic role of E2F8 in cancer [12, 13, 14]. Inhibiting AR synthesis is a unique treatment strategy that has the potential to prevent the development of therapy resistance through the eventual expression of testosterone by the tumor itself and the expression of enzalutamide‐resistant variants.

Marine natural products provide a highly productive resource for the discovery and development of new therapeutics with unique, innovative molecular targets for cancer [15, 16, 17, 18]. We focused our studies on manzamine A (MA), a marine compound isolated from IndoPacific *Acanthostrongylophora* species, an emerging drug candidate with a unique mechanism(s) of action [19, 20]. For the first time, we investigated the anti‐cancer properties of MA targeting prostate cancer *in vitro* and *in vivo* and demonstrated that MA abrogated the AR‐FL and its splice variance AR‐V7 transcription via inhibition of E2F8 function. Thus, blocking the interaction between E2F8 and DNA successfully abrogates AR synthesis and AR‐regulated genes and provides a novel mechanism for controlling treatment‐resistant prostate cancer.

## 2 Materials and methods

### 2.1 Cell culture

Human prostate cancer cell lines LNCaP (CVCL_0395), 22Rv1 (CVCL_1045), PC3 (CVCL_0035), and DU145 (CVCL_0105) were purchased from American Type Culture Collection (ATCC, Manassas, VA, USA) and were maintained in culture media as per instructions. LNCaP cells express *AR*, while the 22Rv1 cells express both *AR* and enzalutamide‐resistant *AR* variant 7 (*AR*‐V7). PC3 cells expressing *AR* (PC3‐AR) were kindly provided by B. Li (University of Kansas Medical Center). All cell lines were maintained in mycoplasm‐free conditions and confirmed for cross‐contamination by short tandem repeat profiling in February 2023 (Labcorp, Burlington, NC, USA).

### 2.2 Analysis of cell viability, growth kinetics, cell proliferation, and colony formation assay

Cell viability was analyzed by plating 1 × 10^4^ cells per well in 96 well plates, and the cells were treated with different concentrations (0–80 μm) of MA for 24, 48, and 72 h against dimethyl sulfoxide (DMSO) control. After the respective time point treatment was completed, the MTT assay was performed, and the cell viability was determined following colorimetric assay as per the manufacturer's instructions. Prostate cancer cells (1 × 10^6^) were seeded in 100 mm tissue culture petri plates for cell growth kinetics. The next day, the cells were fed with the fresh cell culture media containing different concentrations of MA (0, 2.5, 5.0 μm) and were incubated for 24, 48, and 72 h to count the cells using trypan blue assay.

LNCaP, 22Rv1, PC3, and DU145 cells were seeded at a density of 1500 cells per well in six‐well plates and treated with 2.5 and 5.0 μm concentrations of MA for colony formation assay. After 72 h of treatment duration, cells were fed with fresh culture media and were allowed for colony formation. On day 15, cells were washed with phosphate buffer saline (PBS), fixed in ice‐cold Methanol for 15 min at −20 °C, and stained in QC Colloidal Coomassie blue (Cat. no. 1610803; BioRad, Hercules, CA, USA) overnight at room temperature and washed with water. Developed cell colonies were analyzed and counted using imagej software (National Institute of Health, Bethesda, MD, USA).

### 2.3 Western blot analysis and chemicals

Western blot analysis was performed using standard assay using the following primary antibodies (Cell Signaling Technology, Danvers, MA, USA): Cyclin D1: Cat #55506S and E2F8: Cat #34661S (1 : 500), and 1 : 1000 dilution of CDK4: Cat #12790S, p21: Cat #2947S, PARP: Cat #9542S, Caspase‐3: Cat #9661S, AR: Cat #5153S, AR‐V7: Cat #68492S antibodies, and PSA: Cat #ab76113, and hK2: Cat #ab152136 from Abcam (Boston, MA, USA), and their respective secondary antibodies. GAPDH: Cat #3683S/2118S (1 : 8000) or β‐actin: Cat #8457S (1 : 5000) was used as a loading control. Pan‐caspase inhibitor Z‐VAD‐FMK, Enzalutamide, proteasome inhibitor MG132, and protein translation inhibitor cycloheximide were purchased from Selleckchem (Houston, TX, USA).

### 2.4 AR promoter luciferase assay

Co‐author A. Poletti provided the AR promoter luciferase plasmids, which were used to analyze the effect of MA on *AR* transcription activity. These details of plasmids containing a human core promoter and cooperative transcription regulator site are published previously [21]. AR‐negative PC3 and DU145 cells were transfected with human AR promoter luciferase‐expressing plasmid pGL1.8 and pGL2.4, and the promoter activity was determined following luciferase assay kit (Cat no. E1500; Promega, Madison, WI, USA) as per instructions.

### 2.5 mRNA stability assay

Prostate cancer cell lines LNCaP and 22Rv1 were treated with transcription inhibitor Actinomycin D (5 μg·mL^−1^) with or without MA for different time points (1–8 h). Total RNA was isolated, and the synthesized cDNA was used to quantify *AR* and *AR‐V7* mRNA transcript by qRT PCR, assessing the mRNA degradation.

### 2.6 *In vivo* tumor growth study

The animal procedures were approved (AUA6411) by the Institutional Animal Care and Use Committee (IACUC) at the Medical College of Wisconsin. Six‐to‐eight‐week‐old male athymic nude mice (Crl:NU(NCr)‐Foxn1nu) were purchased from the National Cancer Institute and were housed in our clean facility with unrestricted food and water supply. Following acclimatization, 25 mice were inoculated subcutaneously with 0.75 × 10^6^ cells in a 100 μL volume mixed in a 1 : 1 ratio of PBS and Geltrex (Thermo Fisher, Waltham, MA, USA). Mice with palpable tumors were randomized and divided into control and two treatment groups for different dose schedules. Tumor volume and body weight were measured thrice per week using a vernier caliper. The test drug, MA, was resuspended in DMSO and stored in aliquots of 100 μL each at 20 °C until administration by oral gavage. On the day of treatment, 100 μL aliquot of MA was diluted with 900 μL of 0.9% NaCl solution. Treatment groups of mice received MA 30 mg·kg^−1^ per BW by oral gavage thrice (*n* = 10) or twice (*n* = 9) weekly for 3 weeks. The control group of mice (*n* = 6) received a mixed solution of DMSO and NaCl in the same proportion. At the end of the treatment period, animals were euthanized, and blood and tissue samples were collected for further analysis.

### 2.7 RNA‐seq analysis

We performed RNA‐seq analysis on MA‐treated PC3 prostate cancer cells to identify a potential molecular target exhibiting anti‐cancer effects in prostate cancer. For RNA‐seq data analysis, the raw sequence data were trimmed using trim galore (https://github.com/FelixKrueger/TrimGalore), and the sequence reads were aligned to the human reference genome (hg38) using hisat2 with mammalian default parameters [22]. Transcript construction, quantification, and normalization of transcript abundance were performed using stringtie2. Differentially expressed genes (DEGs) between the two test groups were identified using deseq2, and the DEGS with a false discovery rate (FDR) < 0.05 were considered statistically significant [23]. KEGG pathways enriched for DEGs were detected using clusterprofiler [24]. Biological pathway activity was inferred from RNA‐seq data using Gene Set Enrichment Analysis [25].

### 2.8 Molecular docking studies

Based on the RNA‐seq data analysis, we identified E2F8 as a potential molecular target of MA. We selected the E2F8 (PDB ID: 4YO2) protein target, and the docking experiment was carried out as previously described [26]. The docking parameters for E2F8 are: x‐center = 8.575; y‐center = −19.554; z‐center = −5.493; grid box spacing = 1.0 Å; and x‐dimension = 40; y‐dimension = z‐dimension = 25.

### 2.9 Statistical analysis

The statistical analysis for tumor growth study was performed using TumGrowth Package R [27] for *in vivo* tumor growth studies and graphpad prism 9 software (GraphPad Software, LLC, Boston, MA, USA) for comparisons with the Welch *t*‐test.

## 3 Results

### 3.1 Biological effect of MA on prostate cancer cell growth inhibition

First, we evaluated the effect of MA in prostate cancer cell lines (LNCaP, PC3, and DU145) over a wide range of MA concentrations (up to 80 μm) at 24, 48, and 72 h and analyzed the cell viability using MTT assay. MA significantly decreased the cell viability of prostate cancer cells in a dose‐ and time‐dependent manner and determined the IC_50_ values in the range of 3–6 μm up to 72 h. Based on the observed IC_50_ values, MA concentrations of 2.5 and 5.0 μm showed a significant inhibition in prostate cancer cell growth in a colony formation assay in the tested lines LNCaP, 22Rv1, PC3, and DU145 (Fig. S1).

### 3.2 Effect of MA on cell cycle regulating proteins and apoptosis in prostate cancer cells

**Fig. 1:**
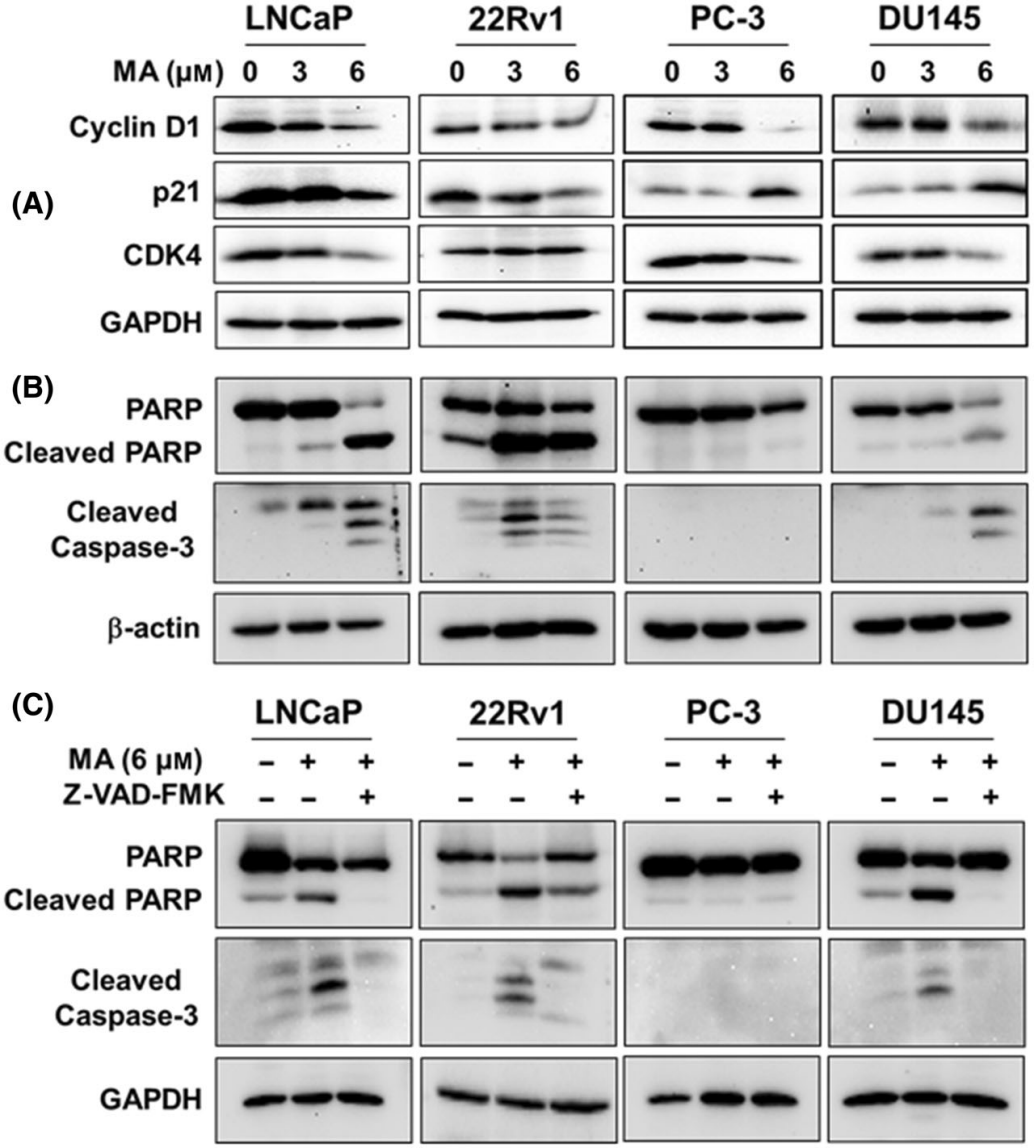
Effect of manzamine A (MA) on cell cycle regulating proteins and apoptosis in prostate cancer cells. Androgen receptor (AR)‐positive (LNCaP and 22Rv1) and AR‐negative (PC3 and DU145) prostate cancer cell lines were treated with MA (3 and 6 μm) for 72 h, and determined for (A) Cyclin D1, p21, and CDK4 protein expression, and (B) Analysis of cleaved PARP and caspase‐3 protein. (C) Prostate cancer cell lines were treated with 50 μm of pan‐caspase inhibitor z‐VAD‐FMK in the presence or absence of MA for 72 h to determine the effect of MA‐induced cleaved PARP and caspase‐3. GAPDH or β‐actin was used as a loading control. A pooled analysis of the data from three independent experiments as a bar graph is shown in Fig. S2.

The loss in control of cell cycle regulation is one of the main drivers of cellular transformation, and inhibition of cell proliferation accompanies changes in cell cycle progression. P21 (WAF1/Cip1) is vital to control cell cycle progression by inhibiting cyclin‐dependent kinase (CDK) functions. We showed that MA significantly suppressed the level of p21 expression in AR‐positive LNCaP and 22Rv1 cells while markedly increased p21 expression in AR‐negative PC3 and DU145 cells compared to control groups (Fig. 1A). It is reported that AR regulates p21 expression in prostate cancer due to the existence of androgen response elements (ARE) in the promoter region of p21 [28]. This observation also indicates that MA might suppress p21 expression by targeting AR in LNCaP and 22Rv1 cells, hence accounting for the differential expression of p21. Similarly, we demonstrated that cyclin D1 expression was significantly reduced following MA treatment compared to the control group in all the tested prostate cancer cell lines (Fig. 1A). A similar effect of CDK4 downregulation was observed in LNCaP, PC3, and DU145 cells; however, MA did not affect the level of CDK4 in 22Rv1 cells. These observations indicated that MA suppressed prostate cancer cell growth and proliferation via inhibiting cell cycle regulatory proteins.

Next, we showed that MA stimulated caspase‐3 cleavage in LNCaP, 22Rv1, and DU145 cells (Fig. 1B). Since all forms of apoptosis involved caspase‐mediated PARP cleavage [29], we verified that MA‐induced PARP cleavage in LNCaP, 22Rv1, and DU145 cells. However, PC3 cells lack the cleavage of caspase‐3 and PARP, indicating a different mechanism of MA action in inhibiting PC3 cell growth. To further validate the action of MA on caspase‐3‐induced apoptosis, the use of pan‐caspase inhibitor Z‐vad‐fmk blocked caspase‐3 with simultaneous inhibition of PARP cleavage in LNCaP, 22Rv1, and DU145 cells, while the PC3 cells remain unaffected (Fig. 1C). A pooled analysis of independent experiments as a bar graph is shown in Fig. S2.

### 3.3 MA‐targeted inhibition of AR and its therapy‐resistant variant AR‐V7

**Fig. 2:**
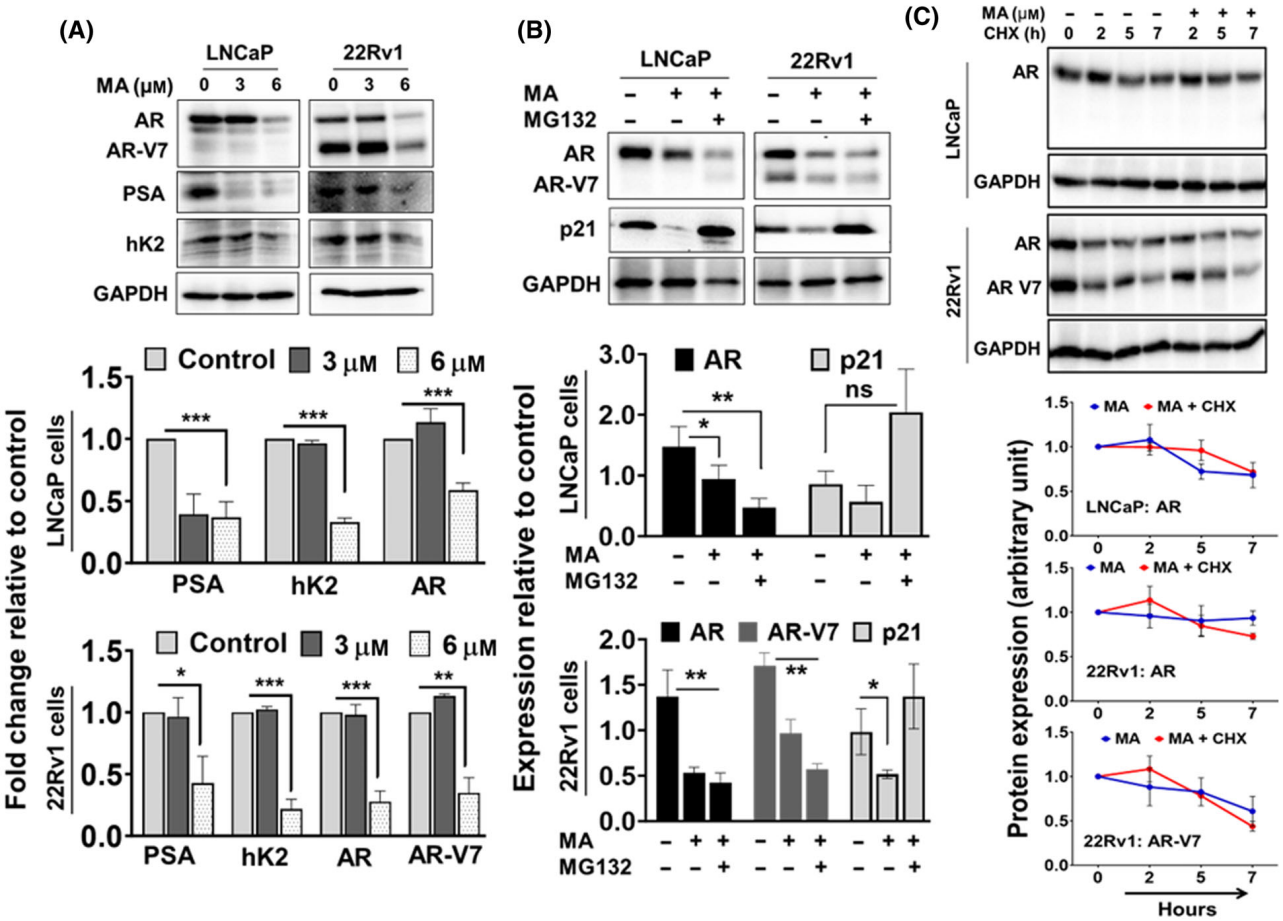
Manzamine A (MA) targeted inhibition of androgen receptor (AR) and its variant AR‐V7. (A) LNCaP and 22Rv1 cells were treated with MA for 72 h, and the protein expression of AR, AR‐V7, PSA, and hK2 was determined by western blot assay. (B) LNCaP and 22Rv1 cells were treated with MA in the presence or absence of proteasome inhibitor MG132 (10 μm), and (C) MA vs MA + cycloheximide (CHX: 100 μg·mL^−1^) for 24 h to analyze the AR degradation. GAPDH was used as a loading control. The bar graph represents the relative expression from three sets of independent experiments as mean ± SE with significance levels at *P < 0.05, **P < 0.01, ***P < 0.001, and ns, non‐significance. All comparisons were performed using Welch *t*‐test.

Since the androgen receptor (AR) and its signaling cascades are prime targets in prostate cancer therapy [30], we focused on the effect of MA on AR regulation. Using AR‐positive prostate cancer cell lines, we showed that MA inhibited AR expression in LNCaP and 22Rv1 cell lines, including AR‐regulated proteins prostate‐specific antigen (PSA) and human kallikrein 2 (hK2), and the AR‐V7 variant in 22Rv1 cells (Fig. 2A). These observations signify the impact of MA inhibiting AR and its regulated signaling in prostate cancer.

Further, to exclude the role of MA on AR protein degradation and instability, LNCaP and 22Rv1 cells were treated with MA in the presence or absence of proteasomal inhibitor MG132 (10 μm). The results showed that MA decreased the AR protein level, and MG132 did not rescue MA‐mediated AR protein suppression in LNCaP and 22Rv1 cells (Fig. 2B). Complementing the observation of proteasome‐independent AR protein suppression, the cycloheximide (CHX: *de novo* protein translation inhibitor) chase assay revealed no differences between AR and AR‐V7 stability in MA and CHX‐treated cells (Fig. 2C). Thus, MA‐induced AR protein repression was independent of the proteasome degradation process.

**Fig. 3:**
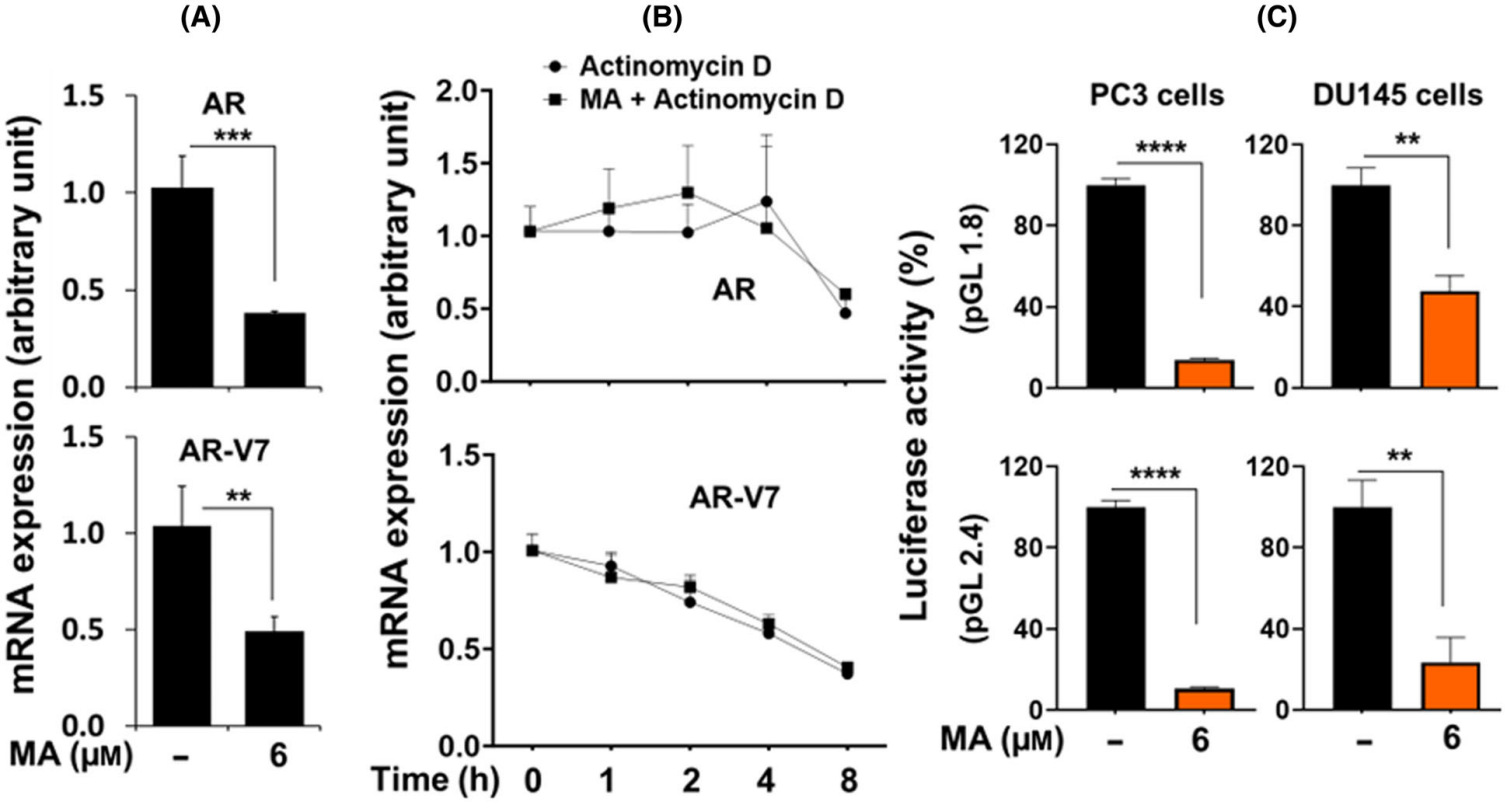
Effect of manzamine A (MA) on androgen receptor (AR) transcription. (A) Effect of MA on suppressing *AR* and *AR‐V7* mRNA transcripts and (B) mRNA stability in the presence or absence of transcription inhibitor Actinomycin D in 22Rv1 cells. (C) Inhibition of luciferase reporter activity of human AR‐luc (pGL1.8 and pGL2.4) in AR‐negative PC3 and DU145 cells following MA treatment. The bar graph represents the relative expression from three sets of independent experiments as mean ± SE with significance levels at **P < 0.01, ***P < 0.001, and ****P < 0.0001 using Welch *t*‐test.

Next, we determined the effect of MA on *AR* mRNA stability in 22Rv1 cells using Actinomycin D (ActD), which inhibits mRNA transcription. Our data showed that MA did not alter the *AR* and *AR‐V7* mRNA stability and that the suppression of *AR* and *AR‐V7* mRNA due to MA treatment was parallel to ActD, supporting the hypothesis that MA blocked the *AR* transcription (Fig. 3A,B). These observations confirmed that MA maintained the integrity of *AR* mRNA and protein *de novo* and the potential mechanism of MA action in regulating AR transcription. To gain further insight into the molecular function of MA on *AR* transcription, we used AR‐negative PC3 cells stably transfected with a plasmid expressing full‐length human AR (pCMV‐AR: PC3‐AR). MA treatment of the PC3‐AR cells for 72 h revealed no effect on the AR protein level (Fig. S3). This observation suggested that the human *AR* core promoter could be a prime target of MA in regulating *AR* transcription. To validate the effect of MA on *AR* transcriptional activity, transfection of AR‐negative PC3 and DU145 cells with luciferase reporter plasmid carrying human *AR* core promoter and subsequent treatment with MA showed inhibition of *AR* promoter activity (Fig. 3C). These results confirmed that MA inhibited *AR* transcription and did not promote AR degradation.

### 3.4 Therapeutic efficacy of MA targeting prostate cancer and organ tissue toxicities

**Fig. 4:**
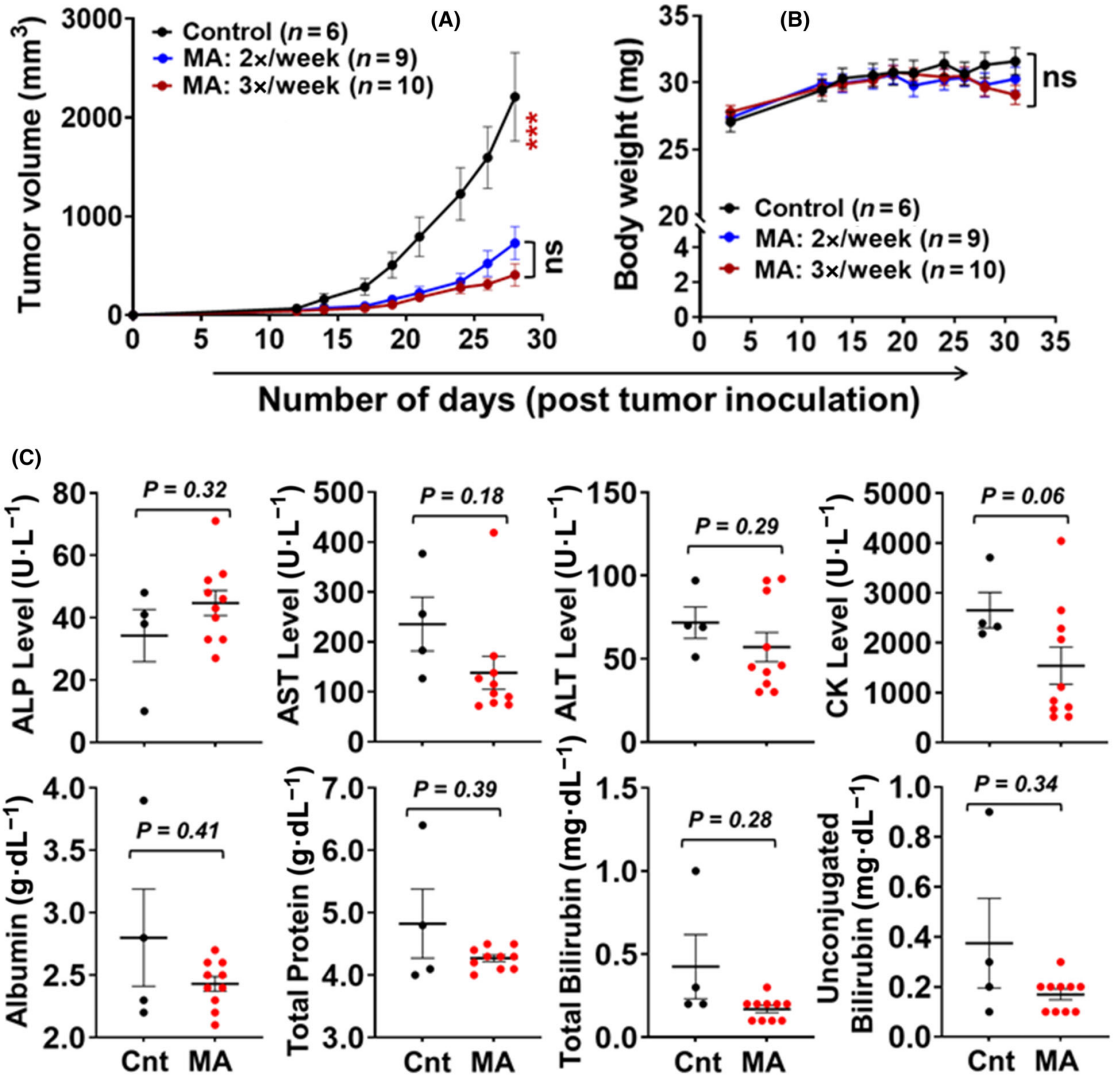
Therapeutic efficacy of manzamine A (MA) suppressing the growth of 22Rv1 cancer cell xenografts. (A) Average tumor growth and (B) Time course of body weight in mice following two different schedules of 30 mg·kg^−1^ dose of MA (*n* = 9 and *n* = 10) compared to vehicle control (*n* = 6). The error bar represents the mean ± SE with significance levels at ***P < 0.001, and ns, non‐significance using spss analysis. (C) Liver panel enzyme and protein activity measured in serum showing the vehicle control (Cnt; *n* = 4) and MA‐treated (*n* = 10) groups of mice. Each dot represents an individual mouse, and the error bar represents the mean ± SE with *P* = values using Welch *t*‐test analysis.

Analyzing the therapeutic efficacy, MA significantly suppressed the enzalutamide‐resistant tumor xenografts of 22Rv1 cells (Fig. 4A). An analysis of variance (ANOVA) of tumor volume at day 31 indicated significant differences in volume between the vehicle control and the two treatment groups (*F*‐test: *P* < 0.0002). These results were further confirmed by the nonparametric Kruskal–Wallis test on tumor volume at day 31 (*P* < 0.0023). The average tumor volume of the vehicle control and MA treatment groups with twice vs thrice per week at 30 mg·kg^−1^ were 2731.1, 905.5, and 575.9, respectively. These results showed a significant difference in tumor volume between the two doses/week at 30 mg·kg^−1^ MA treatment and vehicle control groups (mean difference 1825.6 mm^3^, Bonferroni adjusted *P*‐value < 0.0001) and three doses/week at 30 mg·kg^−1^ treatment and vehicle control groups (mean difference 2155.201 mm^3^, Bonferroni adjusted *P*‐value < 0.0001) but not a significant difference between the dose schedules (twice vs thrice) in the treatment groups (mean difference 329.6 mm^3^, *P* = 0.32).

In a longitudinal analysis, a piecewise linear mixed effects model was fitted to the tumor volume data to ascertain the significance of the changes in volume in the vehicle control and MA treatment groups. A breakpoint was applied on day 17 to capture two different linear phases in tumor growth, day 0–17 and day 18–31. There were no significant differences in tumor volume between the groups in the first phase (days 1–17). However, significant differences in the rate of increase in the average tumor volume between the MA treatment and vehicle control groups were observed in the second phase (days 18–31). The rate of increase in the average tumor volume in the vehicle control groups was 119.3 mm^3^ greater than the two doses/week of the MA treatment group for every passing day from day 18 to day 31 (Bonferroni adjusted *P*‐value 0.0009). The rate of increase in the average tumor volume in the vehicle control group was 143.1 mm^3^ greater than the three doses/week of the MA treatment group per unit increase in time in the second phase (Bonferroni adjusted *P*‐value < 0.0001). There was no significant difference in tumor volume within the MA treatment groups with twice/week or thrice/week (30 mg·kg^−1^) in the second phase (*P*‐value 0.3098).

Following 3 weeks of treatment, MA did not affect the average body weight compared to vehicle control mice at any measured time point during the experiment (Fig. 4B). To assess the potential toxicity of MA, we performed histopathology studies (H & E staining) on major organs, including the heart, spleen, kidney, lung, and liver. There were no histological changes in the heart, spleen, kidney, and lung tissues between the control and MA‐treated groups of mice. No differences were observed between MA‐treated mice and controls as to inflammatory cells or other morphology. No tissue necrosis was seen in either group. However, the MA treatment showed a distinct increase in inflammatory cells in the liver compared to the vehicle control group (Fig. S4). A few neutrophils were noted in the MA‐treated group of mice, but none were noted in the control group. To further investigate the potential toxic effect of MA to the liver, we performed blood chemistry on serum samples (www.idexxbioanalytics.com). The liver panel profile includes alanine aminotransferase (ALT), alkaline phosphate (ALP), aspartate aminotransferase (AST), albumin (ALB), bilirubin (conjugated), bilirubin (unconjugated), bilirubin (total), gamma‐glutamyltransferase (GGT), creatine kinase (CK), and total protein (TP). We found no significant differences in liver analytics between the vehicle control and MA‐treated groups of mice (Fig. 4C). The lipemia index was normal in all the diluted serum samples.

### 3.5 Identification of E2F8 as a molecular target of MA and a regulator of AR

Investigating the molecular mechanism of MA in identifying a potential target and its anti‐cancer effect, RNA‐seq data analysis for the differentially expressed genes (DEGs) showed an upregulation of 118 and a downregulation of 199 transcripts in MA‐treated PC3 cells compared to vehicle control. In addition, the master regulatory gene pathway analysis revealed AR to be one of the top 10 master regulators of ~ 86% of the DEGs. KEGG pathway enrichment analysis suggested that the DEGs were assigned to different categories, where the cell cycle pathway was the most significantly enriched (Fig. S5). A group of down‐regulated genes following MA treatment include ATP‐binding cassette (ABC) subfamily members (*ABCG1* and *ABCA1*), phosphatase and actin regulator (*PHACTR3*), mucin (*MUC2*), G‐protein alpha (*GNA14*), bone morphogenetic protein receptor (*BMPR‐1B*), protein tyrosine phosphatase non‐receptor (*PTPN5*), gastrin‐releasing peptide receptor (*GRPR*), and transcriptional factors (TFs: *E2F2* and *E2F8*). While the functional significance of these genes remains to be investigated, TFs play a vital role in regulating gene expression. Direct or indirect targeting of E2F2 has been associated with prostate cancer [31, 32, 33]. However, we focused on understanding the role of E2F8 in regulating *AR* due to the lack of E2F8 studies in prostate cancer.

**Fig. 5:**
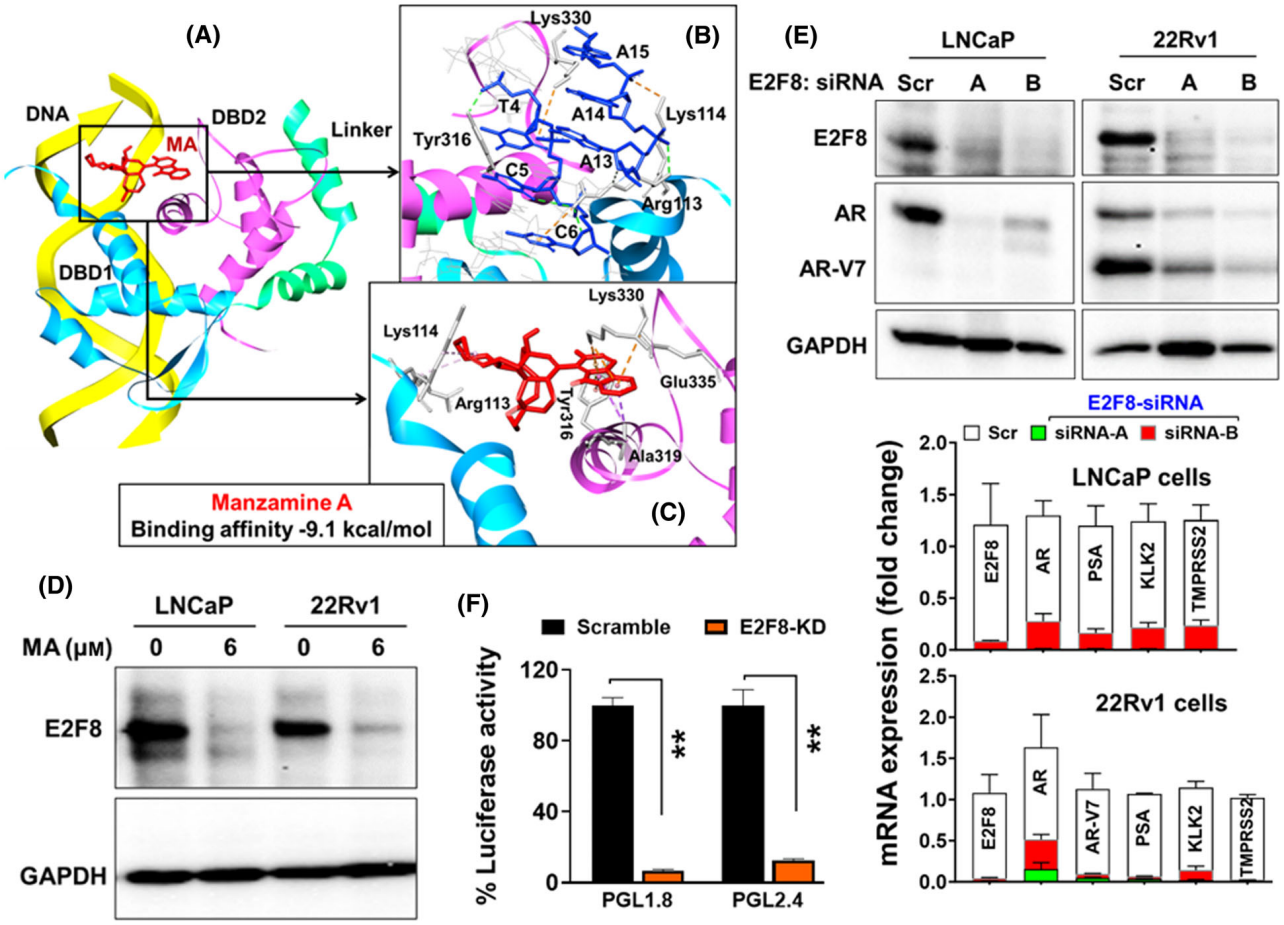
*In silico* molecular docking of manzamine A (MA) with E2F8 using BIOVIA, Dassault Systèmes, Discovery Studio Visualizer v21.1.0.20298. (A) MA (red) and DNA (yellow) is shown in the DNA binding domain (DBD) of E2F8 (PDB ID: 4YO2), which is located between DBD1 (blue) and DBD2 (pink), (B) DNA nucleotides (blue) and (C) MA (red) interactions with the amino acid residues of E2F8. (D) MA‐targeted inhibition of E2F8 in LNCaP and 22Rv1 cells, and the data were repeated twice with similar results. (E) siRNA‐directed E2F8 knockdown and repression of androgen receptor (AR) and its variant (V7) protein (upper panel) and AR‐regulated genes (lower panel). (F) Inhibition of luciferase reporter activity of human AR‐luc (pGL1.8 and pGL2.4) in AR‐positive 22Rv1 cells following siRNA‐directed E2F8 knockdown for 48 h. The bar graphs in E and F represent mean ± SE from three (*n* = 3) independent experiments with significance level at **P < 0.01 using Welch *t*‐test analysis.

Evaluating the possible interaction between the MA and E2F8 as its potential target, *in silico* molecular docking experiment showed that MA docks firmly with the DNA binding domain (DBD) of E2F8 (Fig. 5A). The protein structure of E2F8 consists of two DNA binding domains, i.e., DBD1 and DBD2, connected by a linker [34]. E2F8 binds with DNA at the groove between DBD1 and DBD2 in its role as a transcription factor. The molecular docking showed that MA binds at a similar site as the DNA between DBD1 and DBD2 of E2F8 with a binding affinity of −9.1 kcal·mol^−1^. The molecular docking also revealed that in addition to MA showing binding affinity at the DNA site, it also shared binding interactions with several amino acid residues of E2F8 as the DNA. Both MA and DNA showed binding interactions with Arg‐113 and Lys‐114 with DBD1, and Tyr‐316 and Lys‐330 with DBD2 (Fig. 5B,C). Besides these four amino acid residues, MA was shown to interact with two more amino acid residues on DBD2, i.e., Ala‐319 and Glu‐335, which are located deeper in the binding groove. These observations suggested that MA may potentially inhibit the downstream activity of E2F8 by obstructing DNA binding and subsequent transcription activity.

When analyzing the effect of MA on E2F8, MA‐treated LNCaP and 22Rv1 cells showed inhibition of E2F8 protein (Fig. 5D). Furthermore, the knockdown of E2F8 using two different siRNAs (A & B) abrogated the mRNA and protein expression of AR, AR‐V7, and AR‐regulated *PSA*, *KLK2*, and *TMPRSS2* genes in LNCaP and 22Rv1 cells, suggesting that E2F8 functions in promoting *AR* transcription (Fig. 5E). These results signify the impact of MA as a potential drug inhibiting *AR* transcription via suppression of E2F8 activity in prostate cancer. Lastly, concomitant transfection of AR‐positive 22Rv1 with luciferase reporter plasmid carrying human *AR* core promoter and siRNA‐directed E2F8 knockdown led to inhibition of *AR* promoter activity (Fig. 5F), further supporting a role for E2F8 in *AR* transcriptional regulation.

## 4 Discussion

Natural products have been a core of drug discovery and development since the beginning of recorded time, where naturally occurring compounds provide highly diverse chemical structures, making them excellent candidates for developing anti‐cancer agents [35, 36, 37, 38]. In this study, we demonstrated that MA, a small molecule inhibitor from a marine sponge [19, 20, 39, 40], represses *AR* transcription via the atypical E2F transcriptional factor E2F8 and inhibits prostate cancer cell proliferation *in vitro* and *in vivo*.

The androgen receptor (AR) drives all stages of prostate cancer by various mechanisms, including AR gene mutation, overexpression, epigenetic modification, altered levels of co‐regulators, or the development of new AR variants [41, 42, 43]. Persistent activation of AR and AR‐mediated cascades signifies CRPC development [42]. Therefore, currently used treatment strategies for anti‐CRPC drug development include anti‐androgens that block the effect of androgens by binding at the AR ligand‐binding domain (LBD) or AR N‐terminal domain (NTD) as effective drugs [5]. However, AR evolved into new AR variants, of which splice variants AR‐V7 and ARv567 are the best characterized AR‐Vs [44]. Both variants lack LBD and lead to ligand‐independent activation, causing major limitations for anti‐androgen therapy, including enzalutamide, abiraterone acetate, apalutamide, or darolutamide [8, 45].

Additional antagonists include certain compounds with similar mechanisms of action targeting AR. Marine compounds, sintokamide A (SINT1) and rhizochalinin (Rhiz) showed anti‐prostate cancer effects by targeting AR [15, 16]. Rhiz functions to re‐sensitize AR‐V7‐positive prostate cancer cells to enzalutamide, while SINT1 inhibited prostate cancer cell growth by binding to the activation function‐1 (AF‐1) region in the NTD of AR. Proteosome‐mediated degradation of AR is also considered an alternate approach to depleting AR [46, 47]. However, the efficacy studies of such approaches remain to be established in targeting CRPC. Nonetheless, AR antagonists aimed to target various regions of AR following different mechanisms, emphasized the ongoing importance of targeting AR.

We observed that the suppression of MA‐regulated AR protein was independent of proteasomal degradation. However, AR expression level could also be regulated by a systemic degradation process [48]. Proteasome‐regulated AR degradation and inhibition of 26S proteasome using MG‐132 increases endogenous AR levels in LNCaP cells [49]. Similarly, p21 expression is shown to be short‐lived and is degraded by the proteasome through ubiquitin‐dependent and ‐independent mechanisms [50, 51]. Thus, the MA‐directed AR repression and rescue of p21 following MA and MG132 indicated two different mechanisms and that p21 regulation could also be independent of AR.

Anti‐cancer activities of MA have previously been described in other cancers [52, 53]. Previously, we reported that MA inhibited casein kinase alpha (CK2α) and its downstream target SIX1 oncoprotein, which is associated with tumorigenesis and invasiveness of multiple cancer types [26]. This is the first report demonstrating the anti‐prostate cancer mechanism of MA action with a potential molecular target and a significant inhibition of xenograft tumor growth. Most cancer drugs showed anti‐cancer effects via controlling cell cycle regulatory proteins or interfering with DNA replication in suppressing cancer cell proliferation. Analysis of PARP cleavage and caspase‐3 activation following MA treatment validated the activation of the apoptotic cascade in various prostate cancer cell lines. However, the lack of apoptotic markers, PARP cleavage, or active caspase‐3 in PC3 cells could be ascribed to defects in ceramide formation [54]. It was interesting to note that MA suppressed the p21 protein level in AR‐positive LNCaP and 22Rv1 cells while upregulated p21 in AR‐negative PC3 and DU145 cells. The p21 (WAF1/Cip1) is an essential factor that controls cell cycle progression by inhibiting cyclin‐dependent kinase CDKs functions [55]. In AR‐positive LNCaP and 22Rv1 cells, a decrease in p21 could be attributed to AR repression due to the MA effect since AR is known to upregulate p21 expression. Since the CDK4 and p21 are negatively associated [56], a consistent decrease in CDK4 level and an increase in p21 in AR‐negative PC3 and DU145 cells may account for reduced cell proliferation. The specific mechanism of MA action regulating the molecular interplay between the CDK4 and p21 warrants further investigation. However, these cellular assays indicated a differential mechanism of MA action in AR‐positive and AR‐negative prostate cancer cells.

Manzamine A treatment resulted in not only a decrease in cell cycle regulating proteins and apoptosis induction but also led to AR abrogation and its regulated gene targets. We identified E2F8 as a reliable molecular target of MA and a novel regulator of AR transcription in prostate cancer. E2F8 has been shown to function as either an oncogene or tumor suppressor depending on cellular context [57]. Whereas E2F8 suppresses tumor development in the murine model of liver cancer [58], E2F8 contributes to the oncogenesis in other cancer types, including breast, cervical, and lung cancer [59, 60, 61]. In prostate cancer, an increased E2F8 level is associated with prostate cancer metastasis, and the patients with a high level of E2F8 had significantly worse overall survival [62]. This study also showed that siRNA‐directed E2F8 knockdown in PC3 and LNCaP cells suppressed cell growth and induced G2/M arrest and apoptosis. Indeed, targeting E2F8 has been suggested as a potential therapeutic strategy for cancer treatment; however, there is no small molecule inhibitor of E2F8. A recent study suggested that an immunosuppressive drug cyclosporin A inhibits E2F8 transcription factor via MELK in prostate cancer [63]. However, as shown in our study, the mechanism of MA regulating AR via E2F8 could be different from that of cyclosporin A. Thus, inhibiting the E2F8‐AR axis through MA may have immense translational potential in the treatment of patients with metastatic CRPC, such as in the development of MA analogs with improved therapeutic index. Our *in vivo* efficacy and toxicity studies of MA revealed a significant inhibition of the CRPC tumor xenografts, and a well‐tolerated dose of 30 mg·kg^−1^ did not induce any histological or morphological changes in major organs in xenograft‐bearing mice. The structure of MA is known for decades, but its development as an anti‐cancer agent has been stymied due to a lack of mechanistic understanding and *in vivo* efficacy studies. Our study revealed an intriguing mechanism by which MA acts to suppress prostate cancer development and supports further preclinical and clinical development of MA as a cancer therapy.

## 5 Conclusions

We demonstrated that targeted inhibition of E2F8 by manzamine A (MA) abrogates AR synthesis, its chemo‐resistant variant AR‐V7, and AR‐regulated gene networks. Identification of E2F8 as a novel regulator of AR biogenesis and transcription provides a new therapeutic target in castration‐resistant prostate cancer. As a small molecule inhibitor of E2F8, MA may serve as a potential lead compound to overcome therapy resistance in prostate cancer caused by AR remodeling.

## Conflict of interest

DK and MTH are co‐inventors on a patent characterizing the manzamines mechanism for its therapeutic use. Income: None.

## Author contributions

DK and MTH contributed to conceptualization and funding acquisition; DK, SD, SG, MS, and Y‐MC contributed to methodology; DK, SD, Y‐MC, SG, and PL contributed to formal analysis and writing—original draft preparation; DK, MTH, and AP contributed to resources; DK, SD, KAI, and MS contributed to data curation; DK, SD, SG, Y‐MC, KAI, PL, AP, MTH, and H‐ZC contributed to writing—review, editing, and scientific discussions; DK contributed to supervision and project administration. All authors have read and agreed to the published version of the manuscript.

## Supporting information


**Fig. S1.** Effect of manzamine A (MA) on prostate cancer cell survival and viability.
**Fig. S2.** Pooled data analysis on the effect of manzamine A (MA) on cell cycle regulating proteins and apoptosis in prostate cancer cells.
**Fig. S3.** Analyzing the effect of manzamine A (MA) on androgen receptor (AR) regulation.
**Fig. S4.** Analysis of liver histology in mice following manzamine A (MA) treatment.
**Fig. S5.** RNA‐seq analysis on DMSO (control) and manzamine A (MA)‐treated PC3 cells.

## Data Availability

All data to support the proposed study is included in this publication.
