# Supplementary material for: Manzamine A reduces androgen receptor transcription and synthesis by blocking E2F8‐DNA interactions and effectively inhibits prostate tumor growth in mice
Source: Mol Oncol. 2024 Apr 11;18(8):1966–79. doi: 10.1002/1878-0261.13637 (PMC11306517; doi:10.1002/1878-0261.13637)

Supplementary Figure Legend

SF 1. Effect of manzamine A (MA) on prostate cancer cell survival and viability. (A) Prostate cancer cell lines LNCaP, PC3, and DU145 were treated with a wide range of MA (1 to 80 µM) for 24, 48, and 72 hours and analyzed the percentage cell viability following MTT assay. Percent viable cells were used to calculate IC_{50} (shown in the inset with their respective time points). (B) Inhibition of prostate cancer cell growth in a colony formation assay. Four prostate cancer cell lines LNCaP, 22Rv1, PC3, and DU145, were treated with 2.5 and 5 µM of MA for 72 hours, and the relative cell survival was analyzed by colony formation assay. Data represented as mean ± SE of three independent experiments, and the significance levels are * $P < 0.05$ and ****$P < 0.0001$.

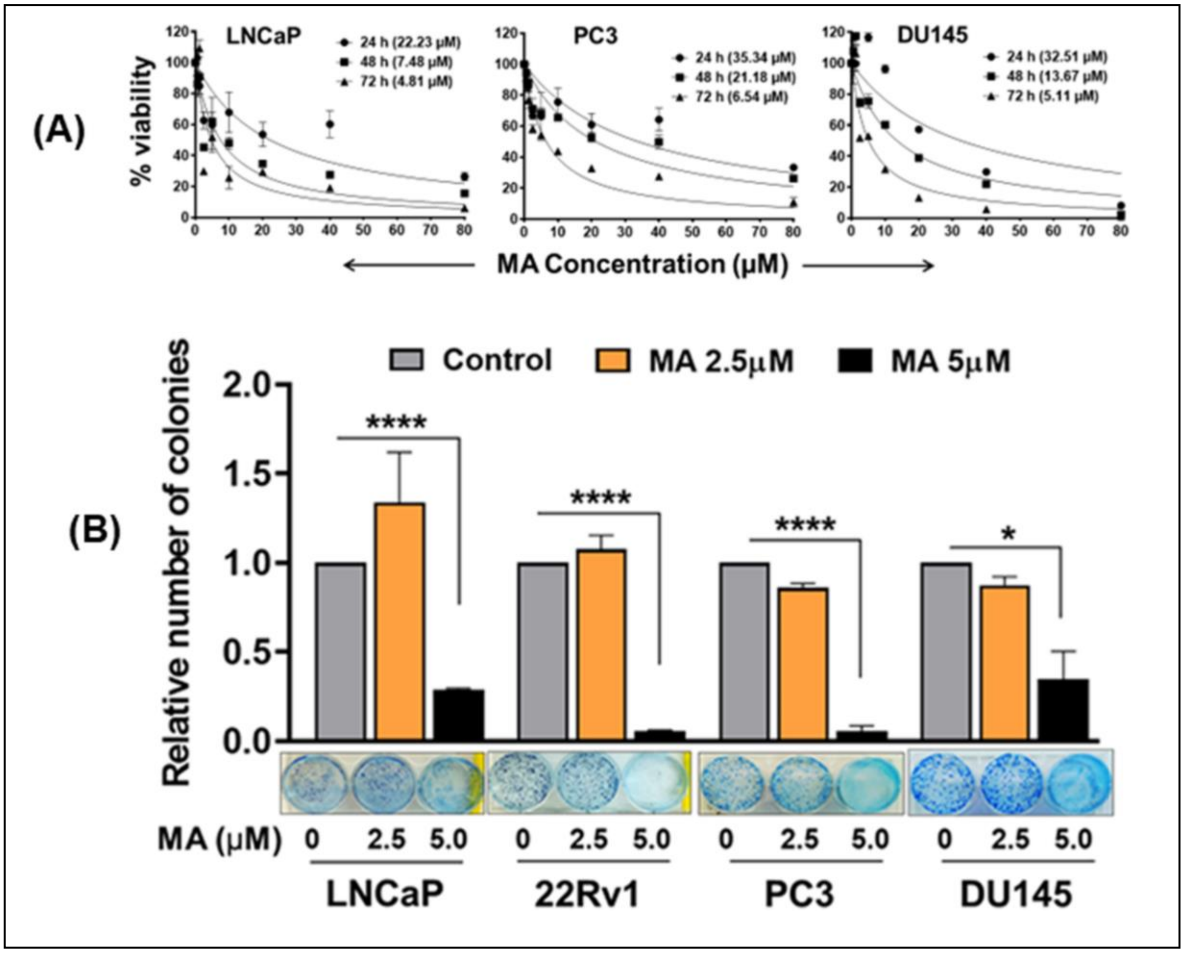

SF 2. Pooled analysis of the data on the effect of manzamine A (MA) on (A) cell cycle regulating proteins and (B) apoptosis in four different prostate cancer cell lines (LNCaP, 22Rv1, PC3, and DU145). The bar graph from three independent experiments in Figure 1 is represented as mean ± SE with significance levels * P < 0.05, ** P < 0.01, ***P < 0.001, and ****P < 0.0001.

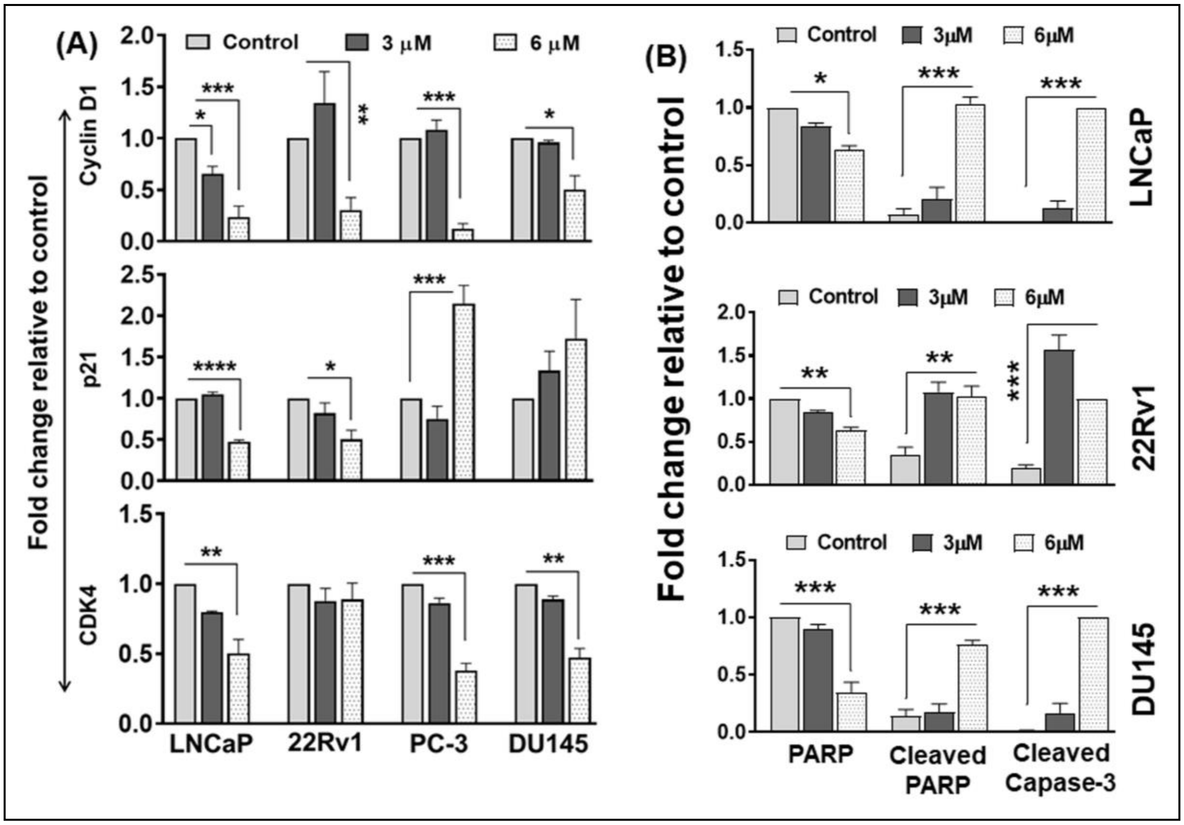

SF 3. Analyzing the effect of manzamine A (MA) on androgen receptor (AR) regulation. AR-negative PC3 cells stably transfected with a plasmid expressing full-length human AR regulated by the CMV promoter (PC3-AR), treated with MA (3 μM and 6 μM) for 72 hours, did not affect the AR protein level.

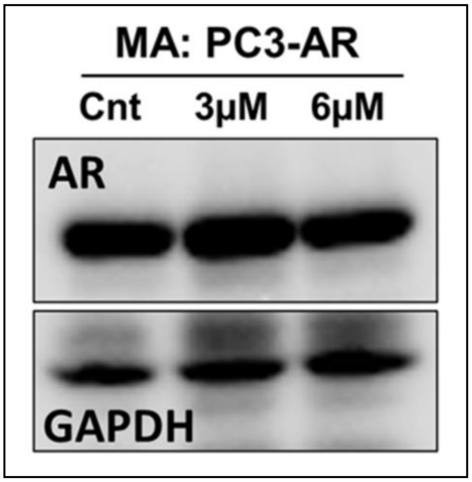

SF 4. Analysis of liver histology in mice following manzamine A (MA) treatment. (A) Representative photomicrographs of H & E stained liver histopathology, with parenchymal inflammatory cells in the MA-treated group of mice compared to vehicle control. Arrows point to two neutrophils. (B) Bar graph of the density of counted inflammatory cells compared between the vehicle control (Cnt; n = 3) and MA-treated (n = 4) groups of mice, represented as mean ± SE with a significant difference (**P < 0.01).

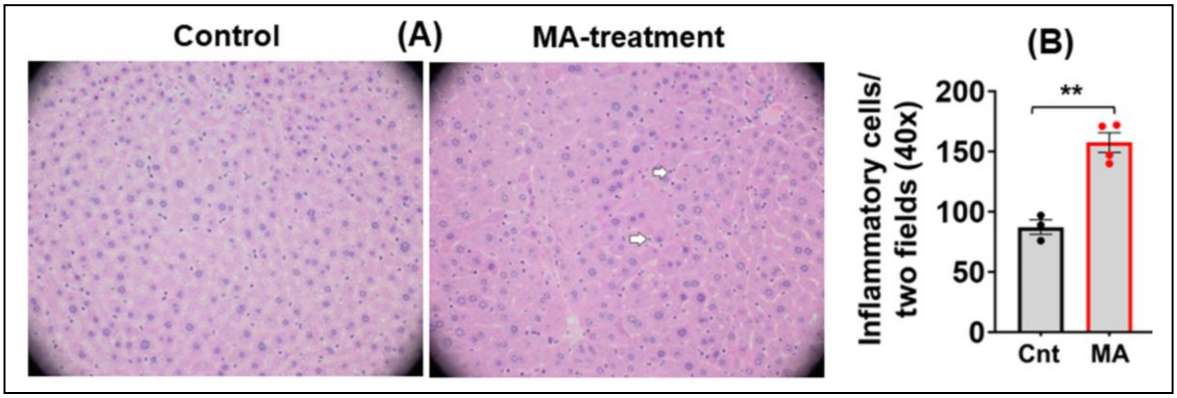

SF 5. RNA-seq analysis on DMSO (control) and manzamine A (MA)-treated PC3 cells. (A) Volcano plot displaying the statistical significance (p-value) versus the relative expression of genes (fold change) between MA-treated and DMSO control PC3 cells. (B and C) Significantly downregulated genes in MA-treated PC3 cells compared to control, involved in different pathways based on KEGG pathway enrichment analysis. (D) Downregulated pathway of cell cycle DNA replication, and the heatmap of downregulated genes in MA-treated cells.

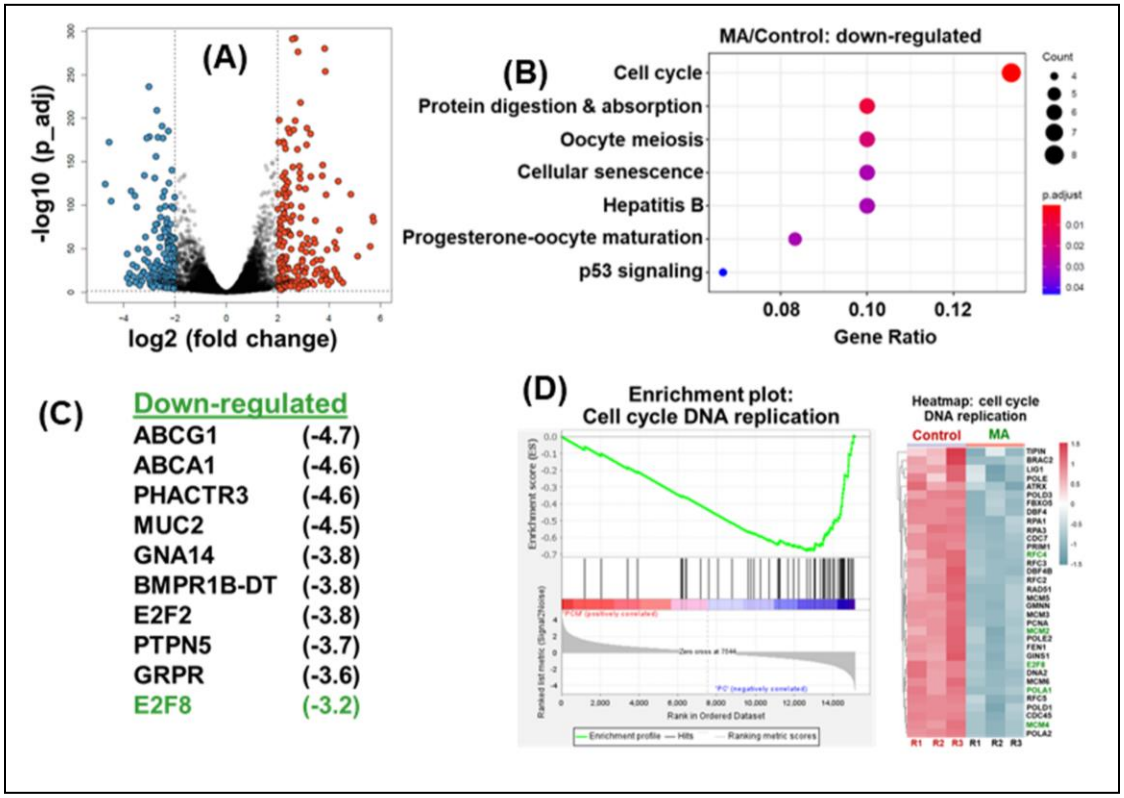

Supplement: Supplementary file 1 — Fig. S1. Effect of manzamine A (MA) on prostate cancer cell survival and viability. Fig. S2. Pooled data analysis on the effect of manzamine A (MA) on cell cycle regulating proteins and apoptosis in prostate cancer cells. Fig. S3. Analyzing the effect of manzamine A (MA) on androgen receptor (AR) regulation. Fig. S4. Analysis of liver histology in mice following manzamine A (MA) treatment. Fig. S5. RNA‐seq analysis on DMSO (control) and manzamine A (MA)‐treated PC3 cells. [file MOL2-18-1966-s001.pdf]
